# Prognostic significance of sealed-off perforation in colon cancer: a prospective cohort study

**DOI:** 10.1186/s12957-018-1530-3

**Published:** 2018-12-04

**Authors:** Kil-yong Lee, Ji Won Park, Inho Song, Ki-young Lee, Sangsik Cho, Yoon-Hye Kwon, Min Jung Kim, Seung-Bum Ryoo, Seung-Yong Jeong, Kyu Joo Park

**Affiliations:** 1 Department of Surgery, Seoul National University College of Medicine, 101, Daehak-ro, Jongno-gu, Seoul, South Korea; 2 Cancer Research Institute, Seoul National University College of Medicine, Seoul, South Korea

**Keywords:** Sealed-off perforation, Free perforation, Colon cancer

## Abstract

**Background:**

Perforated colon cancer is a rare complication, but has a high risk of recurrence. However, most studies have not distinguished sealed-off perforation from free perforation, and the prognosis is unclear. The aim of this study was to evaluate the oncologic outcome of colon cancer with sealed-off perforation.

**Methods:**

Eighty-six consecutive patients who underwent resection for colon cancer with sealed-off or free perforation were included. We defined sealed-off perforation as a colon perforation with localized abscess identified on operative, computed tomography, or pathologic findings, with no evidence of free perforation, including fecal contamination and dirty fluid collection in the peritoneal cavity. Oncologic outcomes were compared between patients with colon cancer with sealed-off perforation and free perforation using a log-rank test and Cox regression analysis.

**Results:**

The sealed-off perforation group included 62 patients, and 24 patients were in the free perforation group. TNM stage and lymphatic, venous, and perineural invasion were similar between the groups. The median follow-up period was 28.9 months (range 0–159). The sealed-off perforation group had better prognosis compared with the free perforation group in terms of progression-free survival (PFS) and overall survival (OS), although there were no statistically significant differences in PFS (5-year PFS 53.7% vs. 40.5%, *p* = 0.148; 5-year OS 53.6% vs. 22.9%, *p* = 0.001). However, in multivariable analysis using the Cox progression test, sealed-off perforation did not show a significant effect on cancer progression (*p* = 0.138) and OS (*p* = 0.727).

**Conclusions:**

Colon cancer with sealed-off perforation showed no difference in prognosis compared with free perforation.

**Electronic supplementary material:**

The online version of this article (10.1186/s12957-018-1530-3) contains supplementary material, which is available to authorized users.

## Introduction

The prevalence of perforation in colon cancer patients is reported to be 3–10% [1–3]. More specifically, colon cancer patients with perforation exhibit a greater frequency of recurrence and poorer overall survival (OS) compared with those without perforation [4, 5]. In the National Comprehensive Cancer Network guidelines, colon cancer with perforation is categorized a high-risk feature [6].

Perforations caused by colon cancer appear as free or sealed-off perforations. A previous study reported that local inflammation affects the prognosis of colorectal cancer [7]; therefore, sealed-off perforation is suspected to exhibit oncological outcomes different from those of free perforation, due to local inflammation caused by the formation of localized abscess. Furthermore, unlike cases of free perforation in which the possibility of tumor dissemination is high, cases of sealed-off perforation prevent tumor spread due to the formation of an abscess cavity. Therefore, sealed-off perforation is expected to have better prognosis compared with free perforation. To the best of our knowledge, no prior study has assessed the prognostic difference in colon cancer patients with free or sealed-off perforation.

This study divided patients into free and sealed-off groups to assess differences in baseline characteristics and whether sealed-off or free perforations have different effects on oncological outcomes.

## Method

Subjects in this study were pathologically diagnosed with primary colon cancer and received surgical treatment at Seoul National University Hospital between March 2002 and August 2017. The inclusion criterion was the presence of either free or sealed-off perforation. The exclusion criteria were history of other malignancy, other accompanying malignancy, and endoscopic or stent-related perforation.

We defined sealed-off perforation as a colon perforation with localized abscess identified on operative computed tomography or pathologic examination, with no evidence of free perforation including fecal contamination and dirty fluid collection in the peritoneal cavity.

Data collected were retrospectively reviewed. Patient clinical characteristics, operative outcomes, pathologic examination results, and postoperative chemotherapy, complications, and mortality were assessed. Complications were classified using the Clavien-Dindo classification. Patients without metastasis were assessed for recurrence while patients with metastasis were assessed for progression.

Study approval was obtained from Seoul National University Hospital Institutional Review Board (IRB).

### Follow-up

The patient was discharged if the following conditions were met: postoperative vital signs were stable, no abnormalities were observed on physical examination, no sign of inflammation was observed on laboratory examination, and no dietary issues were present. Stage II and III patients were asked to return for follow-up visits every 6 months for the first 2 years. To detect potential recurrences in these patients, a carcinoembryonic antigen (CEA) test was performed every 6 months; chest, abdomen, and pelvic computed tomography (CT) were performed annually, and colonoscopy was performed every 6–12 months. Stage IV patients attended regular follow-up visits every 3 months. These patients had CEA every 3 months, CT every 3–6 months, and colonoscopy every year to assess possible recurrence.

### Outcome

The primary end-point of this study was progression-free survival (PFS), and the secondary outcome was local recurrence-free survival (LRFS), peritoneal recurrence-free survival (PRFS), and overall survival. PFS was defined as the time between the date of surgery and the date of diagnosis, cancer progression, or death from any cause. LRFS was defined at the time between the date of surgery and the date of diagnosis of local recurrence, cancer progression, or death from any cause. PRFS was defined at the time between the date of surgery and the date of diagnosis of peritoneal recurrence, cancer progression, or death from any cause. OS was defined as the time period between the date of surgery and the date of death from any cause. When we performed the survival analysis, patients who died within 30 days after surgery were excluded to focus on oncologic outcomes.

### Statistical analysis

To compare baseline characteristics between the free and sealed-off perforation groups, the chi-squared test, Fisher’s exact test, and linear-by-linear association method were used as categorical variables, while the independent *t* test or Mann-Whitney test were used for continuous variables, based on normality of data.

To compare PFS and OS, the Kaplan-Meier method (including log-rank test) was used to perform survival analysis. Cox proportional hazards regression was used to identify independent factors affecting PFS and OS.

Furthermore, the propensity score matching was performed with 1:1 ratio between two groups using logistic regression as estimation algorithm and the nearest neighbor as the matching algorithm. Covariables used in the matching were age, sex, ASA classification, BMI, stage, and postoperative chemotherapy.

Statistical tests were performed using the statistical package for the social sciences (SPSS) version *25.0* for windows (IBM Corp, Armonk, NY, USA), and *p* < 0.05 was considered statistically significant.

## Results

**Fig. 1:**
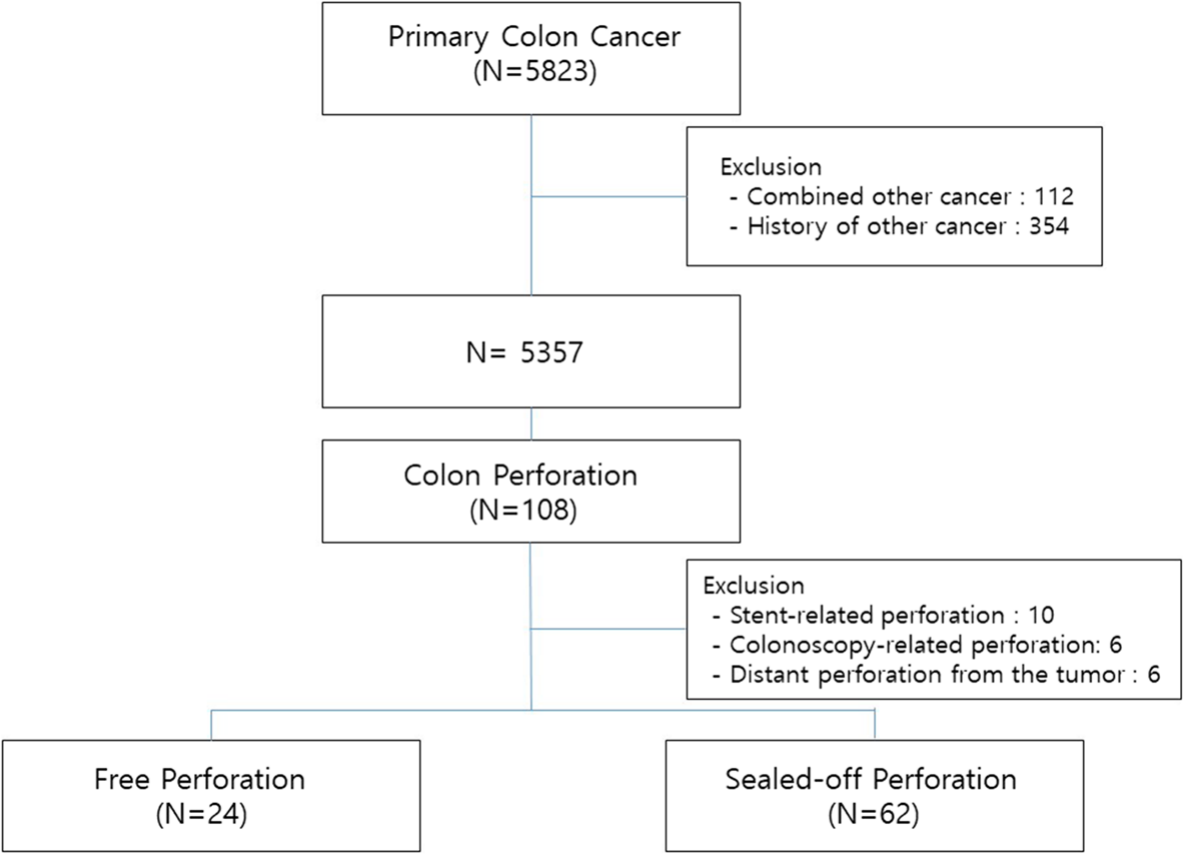
Flow chart of patient selection

Of 5823 patients who received surgical treatment for primary colon cancer at Seoul National University Hospital, 108 had colonic perforation. After excluding patients with perforations caused by stenting or colonoscopy and those with perforations distant from the tumor, 86 patients were included in the final study cohort. Of these, 24 had a free perforation and 62 had a sealed-off perforation (Fig. 1). The median follow-up was 28.9 months (range 0–159 months).

Baseline patient characteristics are shown in Table 1. Patients in the free perforation group were older than those in the sealed-off perforation group (76.1 ± 12.5 vs. 59.4 ± 15.5 years). In addition, the free perforation group had more patients with higher American Society of Anesthesiologists (ASA) class and fewer received postoperative chemotherapy (29.2% vs. 74.2%). Emergency operation was performed more frequently in the free perforation group. (91.7% vs 22.6%, *p* = 0.000). The rate of postoperative complications in the free perforation group was statistically significantly higher than that in the sealed-off perforation group (Table 1). The duration of hospital stay was longer in the free perforation group than in the sealed-off perforation group, but the difference was not statistically significant (17.4 ± 14.2 days vs. 12.0 ± 12.3 days, *p* = 0.062). The 30-day mortality did not differ statistically but was higher in the free perforation group than in the sealed-off perforation group (12.5% vs. 1.6%, *p* = 0.064).

**Table 1:** Baseline characteristics

	Free perforation (*n* = 24)	Sealed-off perforation (*n* = 62)	*p* value
Age (years old)	76.1 ± 12.5	59.4 ± 15.5	0.000
Sex			0.874
Male	14 (58.3%)	35 (56.5%)	
Female	10 (41.7%)	27 (43.5%)	
BMI (kg/m^2^)	22.7 ± 3.5	22.0 ± 2.8	0.458
ASA class			0.001
1	4 (19.0%)	26 (44.8%)	
2	8 (38.1%)	26 (44.8%)	
3	8 (38.1%)	6 (10.3%)	
4	1 (4.8%)	0 (0%)	
Surgery			0.000
Elective	2 (8.3%)	48 (77.4%)	
Emergency	22 (91.7%)	14 (22.6%)	
Operation intent			0.752
Curative	19 (79.2%)	52 (83.9%)	
Palliative	5 (20.8%)	10 (16.1%)	
Tumor differentiation			0.858
Adenocarcinoma, well differentiated	1 (4.2%)	3 (4.8%)	
Adenocarcinoma, moderate differentiated	19 (79.2%)	48 (77.4%)	
Adenocarcinoma, poorly differentiated	3 (12.5%)	8 (12.9%)	
Mucinous carcinoma	1 (4.2%)	2 (3.2%)	
Signet ring cell carcinoma	0 (0.0%)	1 (1.6%)	
Size (cm)	5.8 ± 2.3	6.6 ± 2.3	0.100
pT			0.687
3	12 (50.0%)	28 (45.2%)	
4	12 (50.0%)	34 (54.8%)	
pN			0.779
0	10 (41.7%)	27 (43.5%)	
1	11 (45.8%)	23 (37.1%)	
2	3 (12.5%)	12 (19.4%)	
The number of metastatic LN	1.5 ± 2.9	2.2 ± 3.8	0.325
The number of harvest LN	18.9 ± 9.5	23.3 ± 11.4	0.091
M			0.588
0	16 (66.7%)	45 (72.6%)	
1	8 (33.3%)	17 (27.4%)	
Proximal margin (cm)	27.5 ± 30.2	19.5 ± 20.4	0.088
Distal margin (cm)	13.9 ± 14.5	10.7 ± 9.5	0.395
Lymphatic invasion			0.848
Absent	13 (54.2%)	35 (56.5%)	
Present	11 (45.8%)	27 (43.5%)	
Venous invasion			0.161
Absent	15 (62.5%)	45 (77.6%)	
Present	9 (37.5%)	13 (22.4%)	
Perineural invasion			0.541
Absent	17 (70.8%)	37 (63.8%)	
Present	7 (29.2%)	21 (36.2%)	
Postoperative complication			
(Clavien-Dindo classification)			0.000
No complication	9 (37.5%)	50 (42.5%)	
Grade I	2 (8.3%)	0 (0%)	
Grade II	4 (16.7%)	8 (12.9%)	
Grade III	5 (20.8%)	3 (4.8%)	
Grade IV	4 (16.7%)	1 (1.6%)	
Postoperative chemotherapy	7 (29.2%)	46 (74.2%)	0.000

*BMI* body mass index, *ASA class* American Society of Anesthesiologists classification, *LN* lymph nodes

**Fig. 2:**
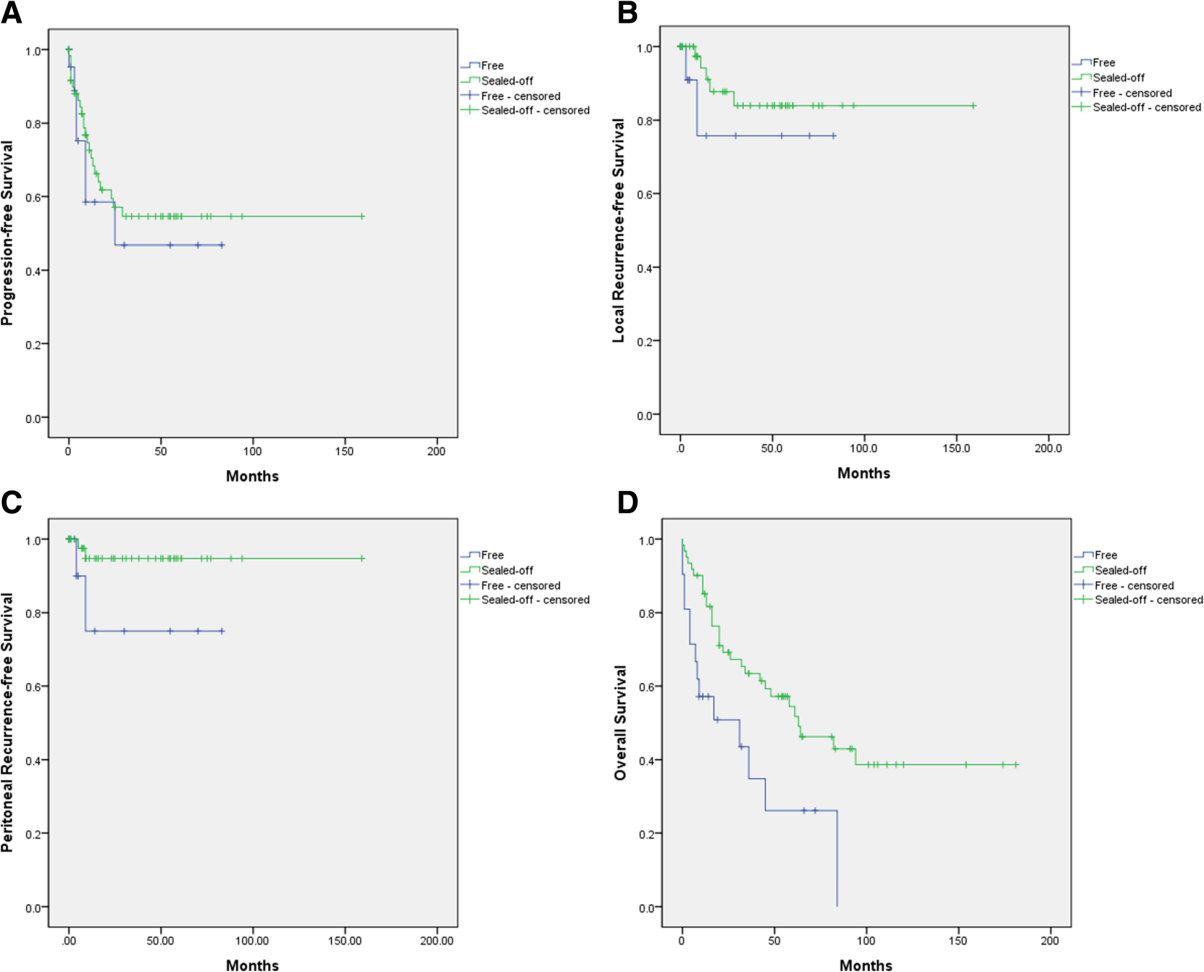
Kaplan-Meier curves between sealed-off (green line) and free perforation (blue line). **a** Progression-free survival. **b** Local recurrence-free survival. **c** Peritoneal recurrence-free survival. **d** Overall survival

The median PFS durations for the free and sealed-off perforation groups were 13.8 months (range 0–83 months) and 27.9 months (range 0–159), respectively, with a 5-year PFS of 40.5% and 53.7%. The sealed-off perforation group displayed a better trend in the Kaplan-Meier curve. However, the log-rank test did not show statistically significant differences between the groups (*p* = 0.148) (Fig. 2b). LRFS and PRFS according to the type of recurrence were analyzed. Five-year LRFS of 75.8% and 84.0% and 5-year PRFS of 75.0% and 94.7% were observed between free and sealed-off perforation groups. The sealed-off perforation group appeared to have a better prognosis in terms of LRFS and PRFS (Fig. 2b, c). However, there was no significant difference between the two groups in the log-rank test (LRFS, *p* = 0.315; PRFS, *p* = 0.069).

The median OS durations for the free and sealed-off groups were 19.6 months (range 0–84) and 49.3 months (range 0–181) respectively, with a 5-year OS of 22.9% and 53.6%. The sealed-off perforation group appeared to have a better prognosis (Fig. 2d). There was a significant difference between the two groups in the log-rank test (*p* = 0.001).

Cox proportional hazards regression was performed to identify the factors associated with cancer progression. Several factors including ASA class 3 (*p* = 0.024), tumor size (*p* = 0.005), and T stage (*p* = 0.043) affected cancer progression, but sealed-off perforation did not show a significant effect on cancer progression (*p* = 0.138) (Table 2).

**Table 2:** Risk factors associated with cancer progression

	Hazard ratio	95% confidence interval	*p* value
Age (years old)	0.986	0.940–1.033	0.545
Sex (female)	2.039	0.677–6.141	0.205
ASA class			0.057
1	1 (reference)	1 (reference)	
2	0.914	0.237–3.523	0.897
3	8.192	1.313–51.120	0.024
BMI (Kg/m^2^)	1.079	0.888–1.310	0.444
Sealed-off perforation	0.443	0.151–1.300	0.138
Emergency operation	0.082	0.008–0.864	0.037
Palliative	2.867	0.423–19.450	0.281
Size(cm)	1.483	1.074–2.046	0.017
pT			0.012
3	1 (reference)	1 (reference)	
4	10.657	1.669–68.028	
pN			0.697
1	0.603	0.170–2.139	0.434
2	0.606	0.096–3.816	0.594
M1	4.594	0.838–25.197	0.079
Proximal margin (cm)	1.041	1.002–1.082	0.038
Distal margin (cm)	0.949	0.887–1.016	0.134
Lymphatic invasion	1.535	0.514–4.568	0.444
Venous invasion	1.382	0.340–5.626	0.651
Perineural invasion	1.193	0.276–5.150	0.813
Postoperative chemotherapy	3.376	0.622–18.322	0.159
Postoperative major complication†	5.628	0.991–31.970	0.051

*BMI* body mass index, *ASA class* American Society of Anesthesiologists classification

†Clavien-Dindo classification grade 3 or higher was defined as major complication

In an analysis to identify risk factors for OS, ASA class (*p* = 0.011) and major postoperative complications (*p* = 0.049) were statistically significant. However, the presence of sealed-off perforation was not a factor affecting OS (*p* = 0.727) (Table 3).

**Table 3:** Risk factors for overall survival

	Hazard ratio	95% confidence interval	*p* value
Age (years old)	1.034	0.998–1.071	0.068
Sex (female)	0.839	0.350–2.012	0.694
BMI (kg/m^2^)	0.927	0.811–1.059	0.265
ASA class			0.011
1	1 (reference)	1 (reference)	
2	0.465	0.16–1.320	0.150
3	3.503	0.977–12.563	0.054
Sealed-off perforation	0.759	0.162–3.554	0.727
Emergency operation	0.694	0.171–2.812	0.609
Palliative	2.340	0.520–10.532	0.268
Size (cm)	0.886	0.724–1.084	0.239
pT			0.025
3	1 (reference)	1 (reference)	
4	3.480	1.174–10.318	
pN			0.546
0	1 (reference)	1 (reference)	
1	1.029	0.430–2.461	0.950
2	2.168	0.538–8.747	0.277
M1	4.766	1.432–15.860	0.011
Proximal margin (cm)	1.009	0.986–1.032	0.440
Distal margin (cm)	1.013	0.972–1.057	0.535
Venous invasion	1.013	0.246–2.199	0.583
Lymphatic invasion	1.541	0.614–3.862	0.357
Perineural invasion	2.225	0.754–6.569	0.148
Postoperative chemotherapy	0.562	0.188–1.677	0.302
Postoperative major complication†	3.931	1.009–15.319	0.049

*BMI* body mass index, *ASA class* American Society of Anesthesiologists classification

†Clavien-Dindo classification grade 3 or higher was defined as major complication

We performed propensity score matching by adjusting for age, sex, ASA class, BMI, stage, and postoperative chemotherapy between the two groups (Additional file 1: Table S1). However, there was no difference in PFS (*p* = 0.604), LRFS (*p* = 0.919), PRFS (*p* = 0.323), and OS (*p* = 0.062) between the two groups (Additional file 2: Figure S A-D). Furthermore, the perforation type did not appear to be a factor affecting PFS (HR 0.565, 95% CI 0.000–1500.805) and OS (HR 12.930, 95% CI 0.131–1053.246) (Additional file 1: Tables S2 and S3).

## Discussion

This study demonstrated that primary colon cancer patients with sealed-off perforation did not have a better prognosis in PFS and OS compared to patients with free perforation, although the Kaplan-Meier curves with log rank test for overall survival showed a statistically significant difference. Perforation type was not a risk factor for PFS and OS although propensity score matching was performed to correct the differences in baseline characteristics between the two groups.

Colon cancer perforation is known to have a poor prognosis, not only because it frequently occurs in advanced stage cancers but also because perforation itself can cause septic complications [2]. More specifically, tumor perforation can cause tumor dissemination, leading to greater recurrence rate and poorer survival [8]. We expected that sealed-off perforation would result in less dissemination of malignant cells into the peritoneal cavity. However, the difference in PFS between the groups was not statistically significant. Even local recurrence and peritoneal recurrence were not different from each other. This may be due to the smaller number of patients in the free perforation group. In addition, patients in the sealed-off perforation group may exhibit tumor dissemination if the abscess cavity bursts during surgery. However, we could not assess possible rupture of an abscess cavity during dissection, which may have led to statistically non-significant outcomes. Furthermore, better trends for LRFS, PRFS, and PFS were observed in the sealed-off perforation group, although the pathologic findings (TNM stage, and lymphatic, venous, and perineural invasion) were not different between the groups, suggesting that perforation type may affect prognosis.

Although several studies reported the effects of inflammation in the local tumor environment [9–11], the effect of sealed-off perforation on local inflammation has not been sufficiently addressed. This study is the first to assess the difference in oncological prognosis between sealed-off perforation and free perforation. Nonetheless, additional studies should be performed to assess the differences in local immunity according to perforation types.

In the analysis of risk factors affecting cancer progression, T stages were a risk factor, but not N stages. We suspect that N stages could not be identified as risk factors because of the small number of enrolled patients. Poorer OS in the free perforation group compared to the sealed-off perforation group is likely due to older age, greater frequency of comorbidities, and postoperative morbidity. Univariable analysis for OS further supports this hypothesis. The baseline characteristics of free perforation patients were older age, greater frequency of comorbidity, and lower frequency of postoperative chemotherapy. Therefore, we could not narrow the difference between two groups even after propensity score matching.

Postoperative chemotherapy was performed significantly more often in the sealed-off perforation group. Relatively higher ASA class and older age of patients in the free perforation group can explain this finding.

There were several limitations in this study. First, the retrospective design may have introduced selection bias and possibly some errors during data extraction. Second, type II error is a possibility since the number of patients with free perforation was smaller than the number with sealed-off perforation.

In conclusion, colon cancer with sealed-off perforation showed no difference in prognosis compared with free perforation even after propensity score matching was performed. Considering the limitations of a small number of patients and unbalanced patient distribution, additional studies with larger cohort should be performed to assess the effect of sealed-off perforation on local immunity.

## Additional files

Additional file 1: **Table S1** Clinicopathologic characteristics after propensity score matching. **Table S2** Risk factors associated with cancer progression after propensity score matching. **Table S3** Risk factors for overall survival after propensity score matching. (DOCX 21 kb)

Additional file 2: Kaplan-Meier curves between sealed-off (green line) and free perforation (blue line) after propensity score matching. (a) local recurrence-free survival. (b) peritoneal recurrence-free survival. (c) progression-free survival. (d) Overall survival. (PNG 51 kb)
