# Supplementary material for: Prognostic significance of sealed-off perforation in colon cancer: a prospective cohort study
Source: World J Surg Oncol. 2018 Dec 4;16:232. doi: 10.1186/s12957-018-1530-3 (PMC6280413; doi:10.1186/s12957-018-1530-3)
Supplement: Supplementary file 1 — Table S1 Clinicopathologic characteristics after propensity score matching. Table S2 Risk factors associated with cancer progression after propensity score matching. Table S3 Risk factors for overall survival after propensity score matching. (DOCX 21 kb) [file 12957_2018_1530_MOESM1_ESM.docx]

|  | Free Perforation  (n=19) | Sealed-off Perforation  (n=19) | P-value |
| --- | --- | --- | --- |
| Age (year-old) | 74.3±11.6 | 43.4± 10.8 | 0.000 |
| Sex  Male  Female | 10 (52.6%)  9 (47.4%) | 10 (52.6%)  9 (47.4%) | 1.000 |
| BMI (kg/m^2^) | 22.8±3.7 | 21.4±2.6 | 0.196 |
| ASA class  1  2  3  4 | 4 (21.1%)  8 (42.1%)  6 (31.6%)  1 (5.3%) | 12 (63.2%)  5 (26.3%)  2 (10.5%)  0 (0%) | 0.008 |
| Surgery  Elective  Emergency | 2 (10.5%)  17 (89.5%) | 48 (68.4%)  14 (31.6%) | 0.000 |
| Operation intent  Curative  Palliative | 15 (78.9%)  4 (21.1%) | 16 (84.2%)  3 (15.8%) | 1.000 |
| Tumor differentiation  Adenocarcinoma,Well differentiated  Adenocarcinoma,Moderate differentiated  Adenocarcinoma,poorly differentiated  Mucinous carcinoma  Signet ring cell carcinoma | 0 (0%)  16 (84.2%)  2 (10.5%)  1 (5.3%)  0 (0.0%) | 1 (5.3%)  14 (73.7%)  4 (21.1%)  0 (0%)  1 (1.6%) | 0.752 |
| Size (cm) | 5.8±2.4 | 7.2±2.3 | 0.078 |
| T  3  4 | 10 (52.6%)  9 (47.4%) | 11 (57.9%)  8 (42.1%) | 0.744 |
| N  0  1  2 | 9 (47.4%)  9 (47.4%)  1 (5.3%) | 9 (47.4%)  5 (26.3%)  5 (26.3%) | 0.380 |
| The number of metastatic LN | 0.8±1.6 | 2.2±3.6 | 0.258 |
| The number of harvest LN | 18.6±9.8 | 25.2±9.8 | 0.046 |
| M  0  1 | 13 (68.4%)  6 (31.6%) | 14 (73.7%)  5 (26.3%) | 0.721 |
| Proximal margin (cm) | 26.0±28.7 | 16.1±17.8 | 0.134 |
| Distal margin (cm) | 15.6±15.8 | 8.7±6.1 | 0.233 |
| Lymphatic invasion  Absent  Present | 11 (57.9%)  8 (42.1%) | 8 (42.1%)  11 (57.9%) | 0.330 |
| Venous invasion  Absent  Present | 11 (57.9%)  8 (42.1%) | 11 (64.7%)  6 (35.3%) | 0.676 |
| Perineural invasion  Absent  Present | 12 (63.2%)  7 (36.8%) | 12 (70.6%)  5 (29.4%) | 0.637 |
| Hospital stay (day) | 15.0±15.8 | 8.7±6.1 | 0.146 |
| 30-days mortality | 2 (10.5%) | 0 (0%) | 0.486 |
| Postoperative complication  (Clavien-dindo classification)  No complication  Grade I  Grade II  Grade III  Grade IV | 8 (42.1%)  2 (10.5%)  4 (21.1%)  3 (15.8%)  2 (10.5%) | 16 (84.2%)  0 (0%)  1 (5.3%)  2 (10.5%)  0 (0%) | 0.025 |
| Postoperative chemotherapy | 6 (31.6%) | 16 (84.2%) | 0.003 |

Supplement table 1. Clinicopathologic characteristics after propensity score matching

BMI, body mass index ; ASA class, American Society of Anesthesiologists classification ; LN, lymph nodes

Supplement table 2. Risk factors associated with cancer progression after propensity score matching

|  | Hazard ratio | 95% confidence interval | P-value |
| --- | --- | --- | --- |
| Age (year-old) | 1.152 | 0.891-1.490 | 0.280 |
| Sex (female) | 1.958 | 0.027-142.574 | 0.759 |
| ASA class  1  2  3 | 1 (reference)  0.002  0.025 | 1 (reference)  0.000-3.536  0.000-279.490 | 0.257  0.101  0.439 |
| BMI (kg/m^2^) | 1.326 | 0.582-3.017 | 0.502 |
| Sealed-off perforation | 0.565 | 0.000-1500.805 | 0.887 |
| Emergency operation | 0.022 | 0.000-3.451 | 0.139 |
| Palliative | 9.893 | 0.002-49824.96 | 0.598 |
| Size(cm) | 3.056 | 0.711-13.135 | 0.133 |
| Stage |  |  | 0.446 |
| 2  3  4 | 1 (reference)  0.031  46.141 | 1 (reference)  0.000-9.222  0.006-349389.4 | 0.231  0.400 |
| Proximal margin (cm) | 1.188 | 0.837-1.687 | 0.335 |
| Distal margin (cm) | 0.840 | 0.629-1.122 | 0.239 |
| Lymphatic invasion | 9.198 | 0.027-3083.179 | 0.454 |
| Venous invasion | 28.016 | 0.666-1178.241 | 0.081 |
| Perineural invasion | 0.358 | 0.002-61.786 | 0.696 |
| Postoperative chemotherapy | 3.439 | 0.086-137.187 | 0.511 |
| Postoperative major complication† | 1.385 | 0.012-164.740 | 0.894 |

BMI, body mass index ; ASA class, American Society of Anesthesiologists classification

†Clavien-Dindo classification Grade 3 or higher was defined as major complication.

Supplement table 3. Risk factors for overall survival after propensity score matching

|  | Hazard ratio | 95% confidence interval | p-value |
| --- | --- | --- | --- |
| Age (year-old) | 10.075 | 0.934-1.237 | 0.313 |
| Sex (female) | 0.069 | 0.005-0.898 | 0.041 |
| BMI (kg/m^2^) | 1.508 | 0.837-2.716 | 0.171 |
| ASA class |  |  | 0.072 |
| 1  2  3 | 1 (reference)  1.489  37.651 | 1 (reference)  0.077-28.769  1.346-1053.246 | 0.792  0.033 |
| Sealed-off perforation | 12.930 | 0.131-1275.544 | 0.275 |
| Emergency operation | 2.691 | 0.079-91.179 | 0.582 |
| Palliative | 4.253 | 0.061-297.262 | 0.504 |
| Size (cm) | 1.001 | 0.670-1.494 | 0.998 |
| Stage |  |  | 0.487 |
| 2  3  4 | 1 (reference)  0.368  2.007 | 1 (reference)  0.034-4.034  0.018-218.673 | 0.413  0.771 |
| Proximal margin (cm) | 1.068 | 0.973-1.171 | 0.168 |
| Distal margin (cm) | 1.007 | 0.950-1.066 | 0.825 |
| Venous invasion | 6.797 | 0.595-77.686 | 0.123 |
| Lymphatic invasion | 0.521 | 0.080-3.397 | 0.496 |
| Perineural invasion | 2.479 | 0.248-24.470 | 0.440 |
| Postoperative chemotherapy | 2.737 | 0.273-27.470 | 0.392 |
| Postoperative major complication† | 1.987 | 0.247-15.990 | 0.519 |

BMI, body mass index ; ASA class, American Society of Anesthesiologists classification

†Clavien-Dindo classification Grade 3 or higher was defined as major complication.
