# Supplementary figures and images for: Prognostic significance of sealed-off perforation in colon cancer: a prospective cohort study
Source: World J Surg Oncol. 2018 Dec 4;16:232. doi: 10.1186/s12957-018-1530-3 (PMC6280413; doi:10.1186/s12957-018-1530-3)

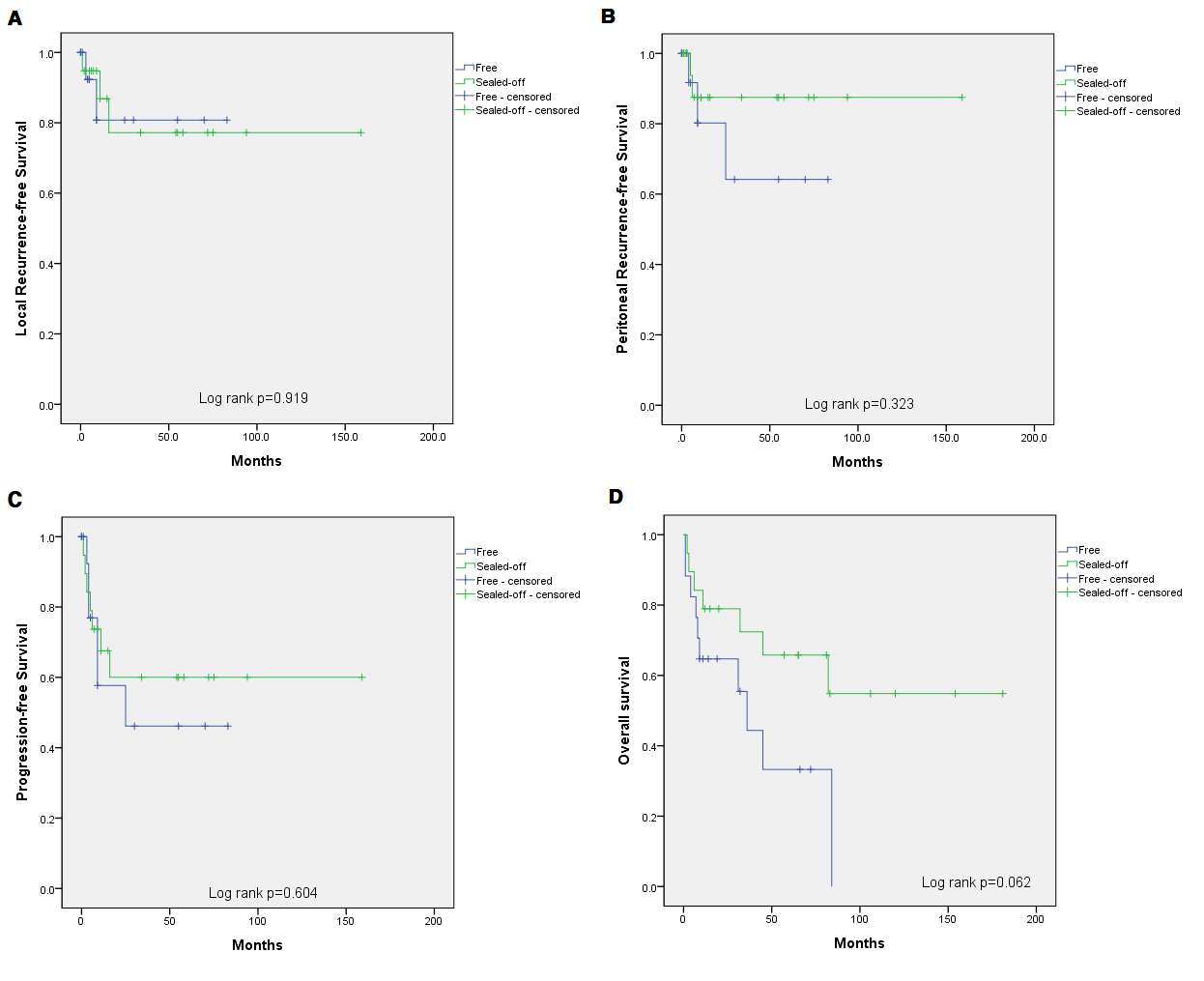

Supplement: Supplementary file 2 — Kaplan-Meier curves between sealed-off (green line) and free perforation (blue line) after propensity score matching. (a) local recurrence-free survival. (b) peritoneal recurrence-free survival. (c) progression-free survival. (d) Overall survival. (PNG 51 kb) [file 12957_2018_1530_MOESM2_ESM.png]
